# Arbitration cancers: analysis from two screening rounds for the Coventry, Solihull and Warwickshire screening service

**DOI:** 10.1186/bcr2964

**Published:** 2011-11-04

**Authors:** S Garnett

**Affiliations:** 1 University Hospital, Coventry, UK

## Introduction

Third-reader screen-reading is undertaken in our unit to reconcile discordant reader results. An audit has been conducted to analyse the nature of cancers diagnosed at assessment for arbitration recalls. The objective was to identify and describe discordant cancer types and appearances.

## Methods

The study period covered two screening rounds. Arbitration records were reviewed and mammographic shape, size, position, cancer type and grade, and histological size were recorded. Both film and digital cases were included and compared. Descriptive statistics were produced comparing discordant and concordant cancers.

## Results

A total of 128 arbitration cancers were analysed (3.6% of total cancers, *n *= 3,516). There were 5,635 total arbitrations of which 27% (*n *= 1,519) were assessed. A total of 8.4% were cancer. There were a higher number of smaller sized (1 to 15 mm) cancers in the arbitrated group, 61% compared with 48% in the concordant group. There was no difference between film and digital cancer size. More cancers appeared as lobular, tubular and DCIS in the arbitration group. There was an equal spread of calcification and mass type mammographic appearances.

## Conclusion

No previous study had specifically analysed arbitration (third-reader) cancers. This audit showed that lesion size is smaller, all cancer types are present and both calcifications and masses are equally represented. Digital cases did not show any smaller cancers for the discordant group. A future audit will be to look at the arbitration interval cancers; that is, when two readers have not recalled a subsequent false positive case, to assess the features that have been ignored.

